# Improving the diagnosis of severe malaria in African children using platelet counts and plasma *Pf*HRP2 concentrations

**DOI:** 10.1126/scitranslmed.abn5040

**Published:** 2022-07-20

**Authors:** James A. Watson, Sophie Uyoga, Perpetual Wanjiku, Johnstone Makale, Gideon M. Nyutu, Neema Mturi, Elizabeth C. George, Charles J. Woodrow, Nicholas P. J. Day, Philip Bejon, Robert O. Opoka, Arjen M. Dondorp, Chandy C. John, Kathryn Maitland, Thomas N. Williams, Nicholas J. White

**Affiliations:** 1 Mahidol Oxford Tropical Medicine Research Unit, Faculty of Tropical Medicine, Mahidol University, Bangkok, Thailand; 2 Centre for Tropical Medicine and Global Health, Nuffield Department of Medicine, University of Oxford, Oxford, UK; 3 KEMRI Wellcome Trust Research Programme, Centre for Geographic Medicine Research, Coast, Kilifi 80108, Kenya; 4 Medical Research Council Clinical Trials Unit, University College London, London, UK; 5 Makerere University, Department of Paediatrics and Child Health, Kampala, Uganda; 6 Department of Pediatrics, Indiana University, Indiana, IN, USA; 7 Institute of Global Health Innovation, Department of Surgery and Cancer, Imperial College, London, UK

## Abstract

Severe malaria caused by *Plasmodium falciparum* is difficult to diagnose accurately in children in high-transmission settings. Using data from 2649 pediatric and adult patients enrolled in four studies of severe illness in three countries (Bangladesh, Kenya, and Uganda), we fitted Bayesian latent class models using two diagnostic markers: the platelet count and the plasma concentration of *P. falciparum* histidine-rich protein 2 (*Pf*HRP2). In severely ill patients with clinical features consistent with severe malaria, the combination of a platelet count of ≤150,000/μl and a plasma *Pf*HRP2 concentration of ≥1000 ng/ml had an estimated sensitivity of 74% and specificity of 93% in identifying severe falciparum malaria. Compared with misdiagnosed children, pediatric patients with true severe malaria had higher parasite densities, lower hematocrits, lower rates of invasive bacterial disease, and a lower prevalence of both sickle cell trait and sickle cell anemia. We estimate that one-third of the children enrolled into clinical studies of severe malaria in high-transmission settings in Africa had another cause of their severe illness.

## Introduction

Severe falciparum malaria is clinically defined as vital organ dysfunction in the presence of circulating *Plasmodium falciparum* parasites (1). The primary objective of this definition is to identify severely ill patients rapidly and provide lifesaving clinical management, notably parenteral artesunate treatment. This definition prioritizes sensitivity over specificity. In areas of moderate and high *P. falciparum* transmission, many apparently healthy children have malaria parasitemia upon examination of their blood by light microscopy or a rapid diagnostic test. Many hospitalized children in high-transmission settings will also be parasitemic but, because major clinical features of severe malaria are not specific, it is difficult to differentiate clinically between severe falciparum malaria caused by extensive sequestration of malaria parasites in the microvasculature (i.e., true severe malaria) (2) and other causes of severe febrile illness accompanied by either coincidental asymptomatic parasitemia or uncomplicated malaria (3, 4). We have shown previously that complete blood counts (platelet counts and total white blood cell counts) provide critical discriminating values in distinguishing true severe malaria from other causes of severe illness (5). To this end, about one-third of a cohort of 2220 Kenyan children diagnosed with severe malaria in a moderate-transmission area were estimated to have had another cause of their severe illness (5). The diagnostic value of complete blood counts was validated using genetic polymorphism data in two different ways: First, the distribution of sickle cell trait (HbAS) correlated very strongly with the estimated probability of severe malaria. The prevalence of HbAS was about five times higher in the subgroup of patients likely to have been misdiagnosed compared with the subgroup that had a likely correct diagnosis (5). Second, we showed that genome-wide false discovery rates could be reduced substantially in a case-control whole-genome association study, in which the output probability weights were used in a “data-tilting” framework to adjust for patient misclassification (5).

The diagnostic value of complete blood counts in severe malaria has great operational utility, because blood counts are widely measured in routine practice at low cost. Moderate thrombocytopenia is a consistent feature of all human malaria infections (6–8), although the diagnostic utility of the platelet count has been debated (9, 10). Platelets are activated in malaria infections and have increased turnover. In severe falciparum malaria, there is endothelial activation with the release of platelet aggregating activated high-multimeric von Willebrand factor from specialized secretory vesicles in endothelial cells (the Weibel-Palade bodies). Platelets may also contribute to and be consumed during parasitized erythrocyte cytoadherence and autoagglutination (11, 12). In addition, severe thrombocytopenia is associated with increased mortality in severe malaria (10, 13, 14). The total white blood cell count is informative, although much less so than thrombocytopenia, with a greater prognostic than diagnostic value, because either very high or very low white cell counts in severe disease are associated with high case fatality ratios (8, 5). Total white counts also vary according to age and ethnicity (15), further confounding cross-study assessments.

The main pathophysiological process in severe falciparum malaria is the extensive sequestration of parasitized erythrocytes in the vascular beds of vital organs (1, 2). These parasites, which cause potentially lethal pathology, have stopped circulating and are not represented in the peripheral blood smear. In African children, the peripheral parasite density is a poor indicator of disease severity and an unreliable diagnostic marker (16). The parasite protein *P. falciparum* histidine-rich protein 2 (*Pf*HRP2), the basis for most rapid diagnostic tests, is liberated mainly at schizont rupture, such that the amount of *Pf*HRP2 released is proportional to the extent of recent schizogony. Plasma *Pf*HRP2 is a much better discriminant of severe falciparum malaria than the peripheral blood parasite count (17, 18). An example of how *Pf*HRP2 can help the interpretation of clinical trial data comes from the large multicenter African Quinine Artesunate Malaria Trial (AQUAMAT), which was a randomized comparison of parenteral artesunate versus parenteral quinine in African children clinically diagnosed as having severe falciparum malaria. Overall, artesunate reduced the mortality by about 22% (19). However, there was evidence for treatment effect heterogeneity across *Pf*HRP2 strata. In the subgroup of children in the highest tertile of *Pf*HRP2, artesunate reduced the mortality by one-third, which is a similar proportion to that observed earlier in the South East Asian Quinine Artesunate Malaria Trial (SEAQUAMAT), a randomized comparison conducted in Southeast Asia (20). In contrast, there was no substantial difference in mortality in the subgroup of children in the lowest tertile of *Pf*HRP2, which suggests that this group was likely to have other causes of severe illness (18).

Defining optimal cutoff values for diagnostic markers in severe malaria is difficult, because there is no gold standard for diagnosis against which to calibrate thresholds in high-transmission areas. In the absence of a gold standard, latent class models can be used to assess the sensitivity and specificity of threshold values for diagnostic indices (21). Latent class models typically rely on having multiple markers measured in the same individuals across different populations with varying disease prevalences, thus allowing for triangulation (21, 22). We applied Bayesian parametric latent class models to admission platelet counts and plasma *Pf*HRP2 concentrations in combination to estimate their diagnostic operating characteristics and to estimate the proportion of misdiagnosed patients in studies of severe malaria. We used data from four prospective studies of severe malaria or severe illness from Uganda, Kenya, and Bangladesh, reflecting a range of *P. falciparum* transmission intensities from high to low and thus a range of false diagnosis rates. Our results suggest that the high rates of severe falciparum malaria misdiagnosis could be reduced substantially by incorporating measurement of platelet counts and plasma *Pf*HRP2 concentrations in the diagnostic criteria.

## Results

### Platelet counts and *Pf*HRP2 concentrations in severe febrile illness

We pooled individual patient data from 2649 severely ill African children and Asian adults, for whom platelet counts and measured plasma *Pf*HRP2 concentrations were available. The patients were from four separate studies in three countries: (i) an observational study of severe falciparum malaria in Bangladesh (*n* = 172; all patients were clinically diagnosed with severe falciparum malaria), (ii) the Ugandan sites of the Fluid Expansion as a Supportive Therapy (FEAST) trial (*n* = 567; a randomized controlled trial of fluid resuscitation approaches in severe childhood illness not specific to severe malaria) (23), (iii) an observational study of cerebral malaria and severe malarial anemia in Kampala, Uganda (*n* = 492) (24), and (iv) a large cohort of children diagnosed with severe malaria in Kilifi, Kenya (*n* = 1418) (25). Malaria transmission intensity is generally low in Bangladesh, moderate in Kilifi and Kampala, and high around the other Ugandan sites.

In total, 27 patients (1%: 1 in Bangladesh, 8 in the FEAST sites in Uganda, and 18 from Kenya) had no detectable *Pf*HRP2 in the enzyme-linked immunosorbent assay (ELISA) but had a parasite density >1000/μl by microscopy. These could have been either assay errors or parasites with *HRP2/3* gene deletions. They were removed from the analysis, leaving a total of 2622 samples. A summary of the patient characteristics in the analyzed dataset is shown in Table 1.

Log_10_-transformed platelet counts and log_10_-transformed *Pf*HRP2 concentrations were strongly inversely correlated in both studies of severely ill African children (ρ= −0.54) and in Bangladeshi adults with severe malaria (ρ= −0.36). Patients with platelet counts in the normal range (>150,000/μl) generally had low to nonmeasurable plasma *Pf*HRP2 concentrations [median concentration was 269 ng/ml; interquartile range (IQR): 24 to 1043], whereas patients with thromobocytopenia (≤150,000/μl) had a median *Pf*HRP2 concentration of 3031 ng/ml (IQR: 1261 to 6035).

### Discriminative value of platelet counts and plasma *Pf*HRP2 in patients diagnosed with severe malaria

**Fig. 1:**
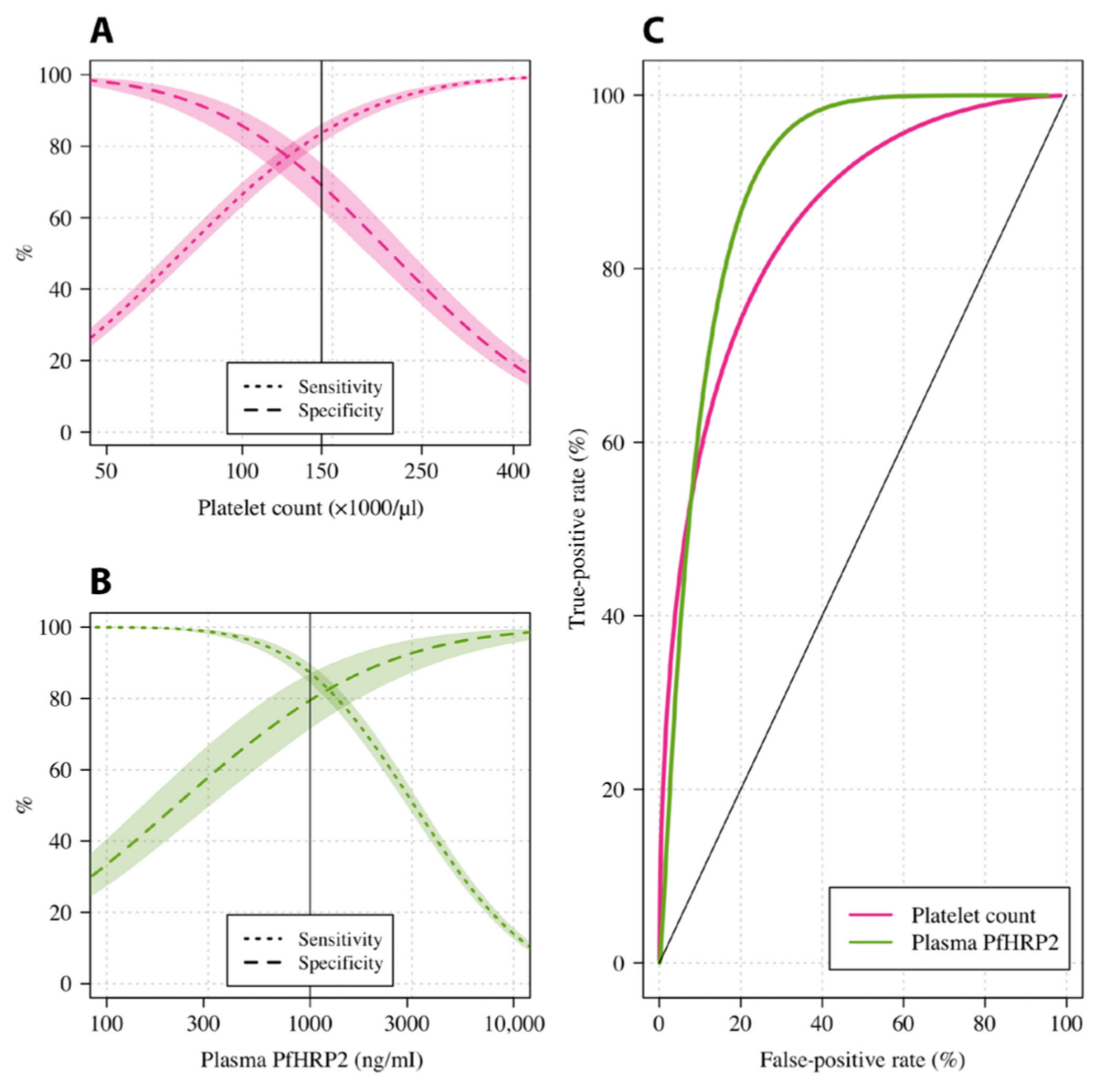
The diagnostic value of platelet counts (pink) and plasma *Pf*HRP2 concentrations (green) estimated using data from 2063 patients diagnosed with severe falciparum malaria in three studies. (**A**) and (**B**) show the mean estimated sensitivity (dotted lines) and specificity (dashed lines) functions for platelet count and plasma *Pf*HRP2, respectively. For the platelet count, thresholds correspond to upper limits, whereas for the *Pf*HRP2 concentration, the thresholds correspond to lower limits. Shaded areas show 95% CI. (**C**) shows the ROC curves for each marker.

We assessed the diagnostic value of platelet counts and plasma *Pf*HRP2 concentrations in patients who were clinically diagnosed with severe malaria (*n* = 2063). This analysis excluded patients from the FEAST trial, which explicitly included nonmalarial causes of severe febrile illness. We fitted a two-component parametric Bayesian latent class model, with the two latent classes representing “severe malaria” and “not severe malaria,” using log_10_-transformed markers (see Materials and Methods for description of the informative priors used and the key assumptions). Figure 1 (A and B) shows the model-estimated sensitivities and specificities for all cutoff values of the platelet count and the *Pf*HRP2 concentration. As expected, the platelet count and the *Pf*HRP2 concentration both had high discriminative value, illustrated by the receiver operating characteristic (ROC) curves (Fig. 1C). Overall, for thresholds giving the same sensitivity, the plasma *Pf*HRP2 concentration had a higher specificity. For example, in these populations, a lower limit of 1000 ng/ml for the *Pf*HRP2 concentration had an estimated sensitivity of 87% and a specificity of 83%. In comparison, an upper limit for the platelet count of 150,000/μl had an estimated sensitivity of 83% but a specificity of 71%. A series of sensitivity analyses (using noninformative priors, using different choices for the parametric model, or using only data from the two studies of African children with severe malaria) showed near-identical results (fig. S1).

### Joint diagnostic thresholds for platelet counts and plasma *Pf*HRP2

For clinical and epidemiological studies, and in contrast to clinical practice, specificity is usually more important than sensitivity. For example, interpretation of effect sizes in randomized trials will depend on the included population and, thus, the specificity of the inclusion criteria. We used the two markers in combination to improve the precision of the definition of severe malaria to achieve low false-positive rates. Under the model, multiple combinations of the platelet count and *Pf*HRP2 concentration have approximately the same operating characteristics for diagnosis, so the optimal choice is subjective and can be made based on operational simplicity (fig. S2). In severe illness with clinical features consistent with severe malaria, the combination of platelet counts of ≤150,000/μl and plasma *Pf*HRP2 concentrations of ≥1000 ng/ml had an estimated diagnostic specificity of 93% and a sensitivity of 74%. If this definition was applied to a hospital cohort with a prevalence of severe malaria of 60%, which is the prevalence estimated for the Kenyan cohort of children with clinically diagnosed severe malaria, we would expect >94% of the resulting population identified by these two markers to have true severe malaria (positive predictive value).

### Estimating the probability of severe malaria

**Fig. 2:**
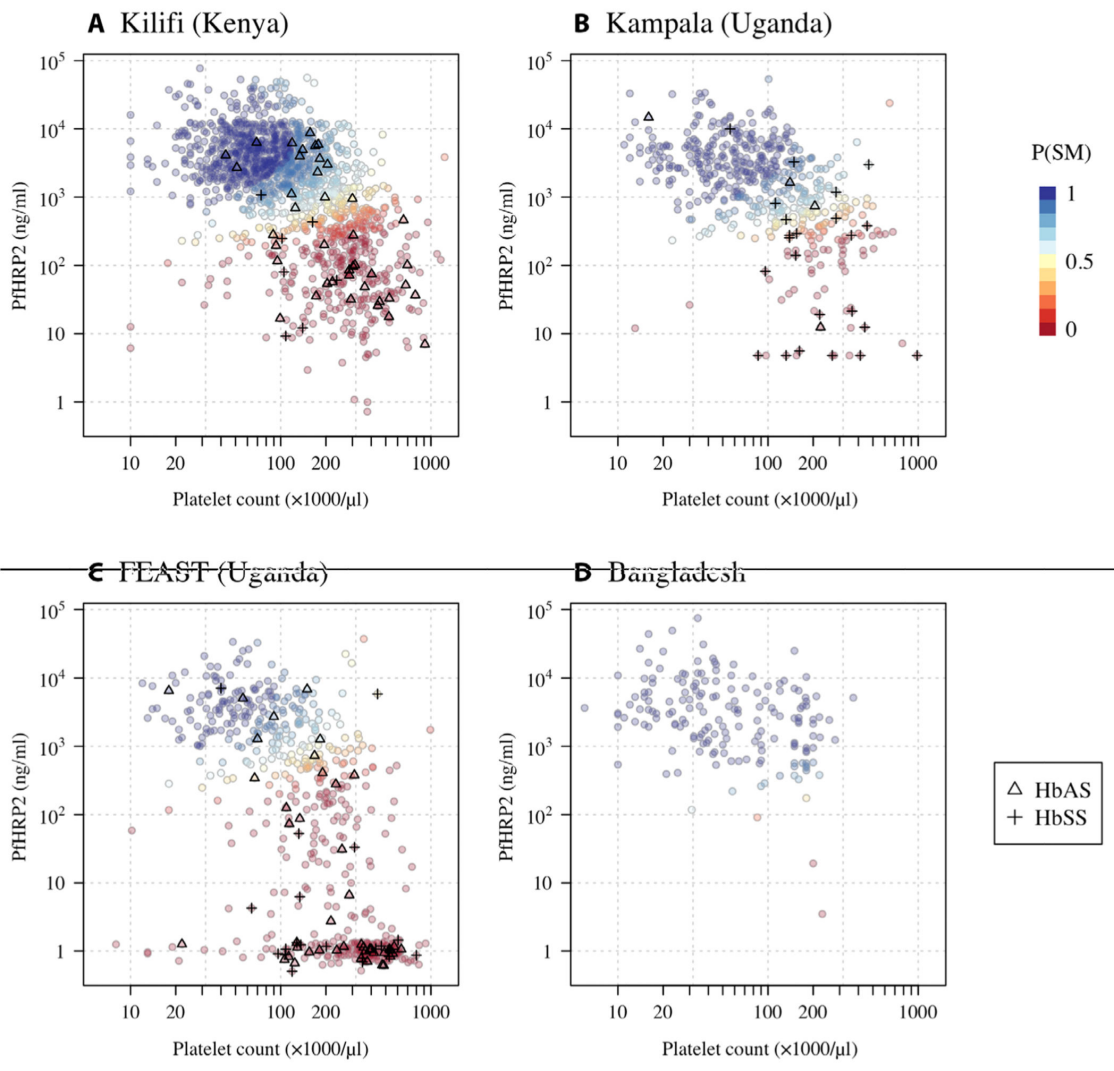
Probabilistic model of severe falciparum malaria using platelet counts and plasma *Pf*HRP2 concentrations in 2622 severely ill patients based on a Bayesian parametric latent class model with three latent classes (a severe malaria class and two not severe malaria classes). (**A**) to (**D**) show the individual data for each study [(A) Kilifi cohort, (B) Kampala cohort, (C) FEAST trial, and (D) Bangladesh cohort]. The colors correspond to the probability of severe malaria under the model (dark blue: high probability; dark red: low probability). Triangles show the individuals with HbAS; crosses show the individuals with HbSS. To show data points with nonmeasurable plasma *Pf*HRP2, nonmeasurable concentrations were set to 1 ng/ml ± random jitter on the log_10_ scale (about half the lower limit of quantification of the assay). P(SM), Probability(Severe Malaria).

We fitted a three-component parametric Bayesian latent class model to all available platelet count and *Pf*HRP2 concentration data from patients from the four studies of severe febrile illness (*n* = 2622 patients) to estimate the individual patient probabilities that severe malaria was the true cause of their severe illness and to calculate the prevalences of true severe malaria among those diagnosed. The additional third component captured a cluster of patients in the FEAST trial who had no detectable plasma *Pf*HRP2 and normal platelet counts (Fig. 2). Under this model, we estimated that the prevalence of true severe malaria was 96% [95% credible interval (CI): 91 to 99] in Bangladeshi adults, 37% (95% CI: 31 to 42) in the Ugandan children enrolled in the FEAST trial, 74% (95% CI: 67 to 79) in the children enrolled in Kampala, Uganda (24), and 66% (95% CI: 61 to 70) in the children diagnosed with severe malaria in Kilifi, Kenya. We note that the FEAST trial intentionally enrolled severely ill children with and without malaria, although 66% of all patients had a diagnosis of severe malaria (23).

Figure 2 shows scatterplots of the platelet counts versus the plasma *Pf*HRP2 concentrations that are colored by the probability of a patient having severe malaria and grouped by study. Dark blue represents a high probability of severe malaria, and dark red represents a low probability of severe malaria. The model estimated a geometric mean platelet count in severe malaria across the four studies of 74,000/μl (95% of patients are predicted to have platelet counts between 17,000 and 312,000) and a geometric mean *Pf*HRP2 of 3135 ng/ml (95% prediction interval: 402 to 24,452). The model-based probabilities of severe malaria were highly concordant with our previously published model that used platelet counts and total white blood cell counts (ρ = 0.64; fig. S3) (5).

We compared the estimated false diagnosis rates for cerebral malaria versus severe malaria without coma and for severe malarial anemia versus severe malaria without severe anemia, where severe anemia is defined as a hematocrit ≤15% in the two cohorts of children clinically diagnosed with severe malaria (table S1). In the Kenyan cohort, the estimated false diagnosis rate was slightly higher in the cerebral malaria group relative to the noncerebral malaria groups: 36% of patients with coma and 26% of patients without coma were classified as “falsely diagnosed” with severe malaria (*P* = 0.001). For the subgroup with severe anemia, the false diagnosis rate was 15%, compared to 36% in the subgroup without severe anemia. In the Ugandan cohort, both of these trends were reversed. A false diagnosis of severe malaria was estimated for only 12% of patients with coma but 40% of patients without coma. For severe anemia, these proportions were 36 and 15%, respectively (the Ugandan cohort only recruited patients with either severe malarial anemia or patients with cerebral malaria).

**Fig. 3:**
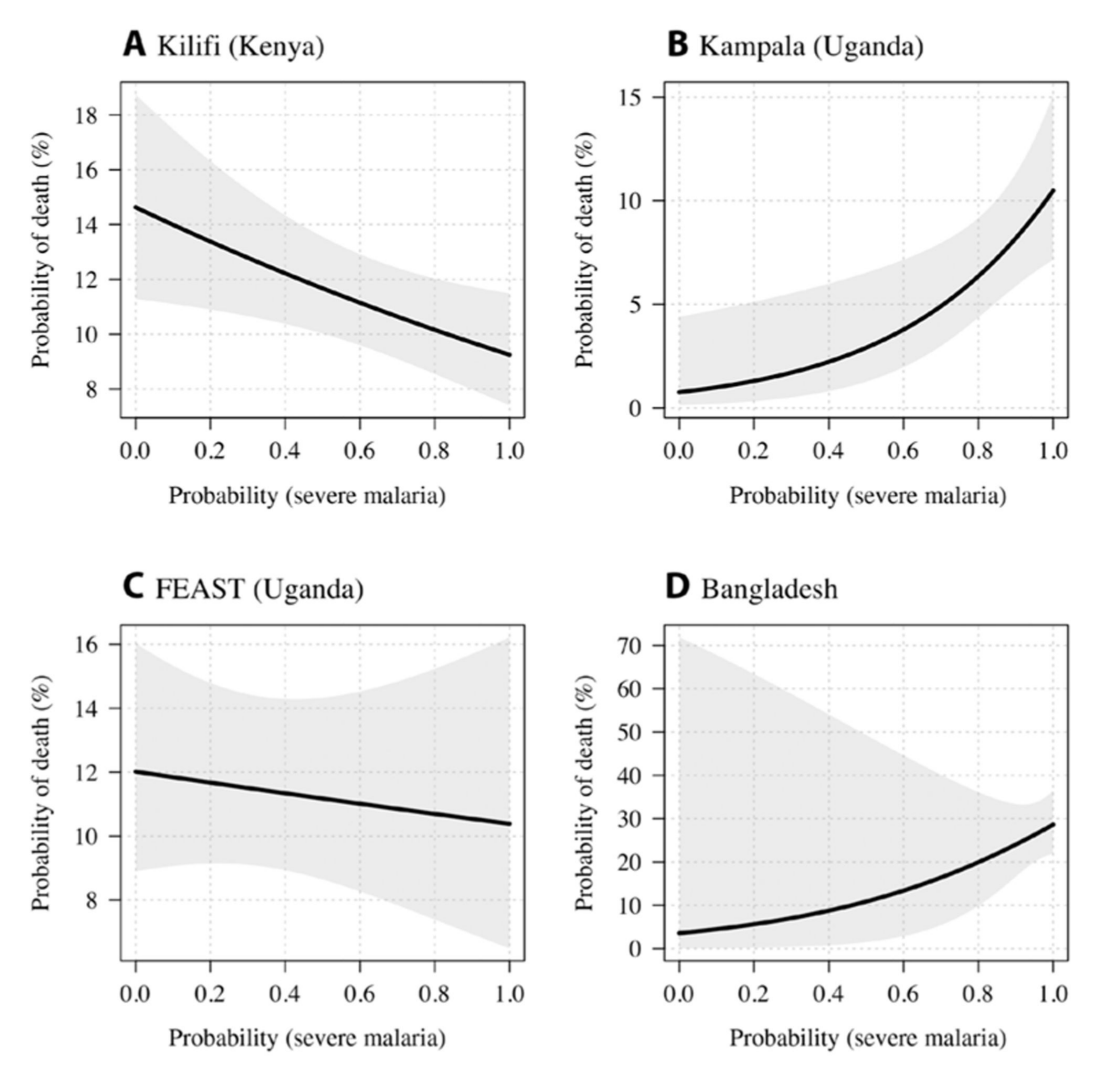
Mortality as a function of the probability of having severe malaria under the Bayesian latent class model (based on platelet counts and *Pf*HRP2 concentrations). The lines (shaded areas) show mean (95% CIs) mortality estimates from logistic regression fits. (**A**) to (**D**) show the individual data for each study [(A) Kilifi cohort, (B) Kampala cohort, (C) FEAST trial, and (D) Bangladesh cohort].

Mortality rates varied substantially as a function of the estimated probability of severe malaria and across the studies (Fig. 3). In African children with a high probability of having severe malaria, the mean mortality was consistently ~10% for the three studies included (Fig. 3, A to C). Apart from the Bangladeshi adults (Fig. 3D), all patients were initially treated with intravenous quinine. The severe malaria mortality in adults from Bangladesh was substantially higher (~30%), consistent with previous studies (20). In Kenyan children (Fig. 3A), the mortality in the misclassified group of patients was higher than in the correctly classified patients (14 versus 10%), whereas the trend was reversed in the Ugandan study (1 versus 10%; Fig. 3B). This is largely explained by the study populations, such that the group with a low probability of having severe malaria in the Ugandan study was predominantly composed of patients with severe anemia without other features of severity.

### Relationship with other admission variables

**Fig. 4:**
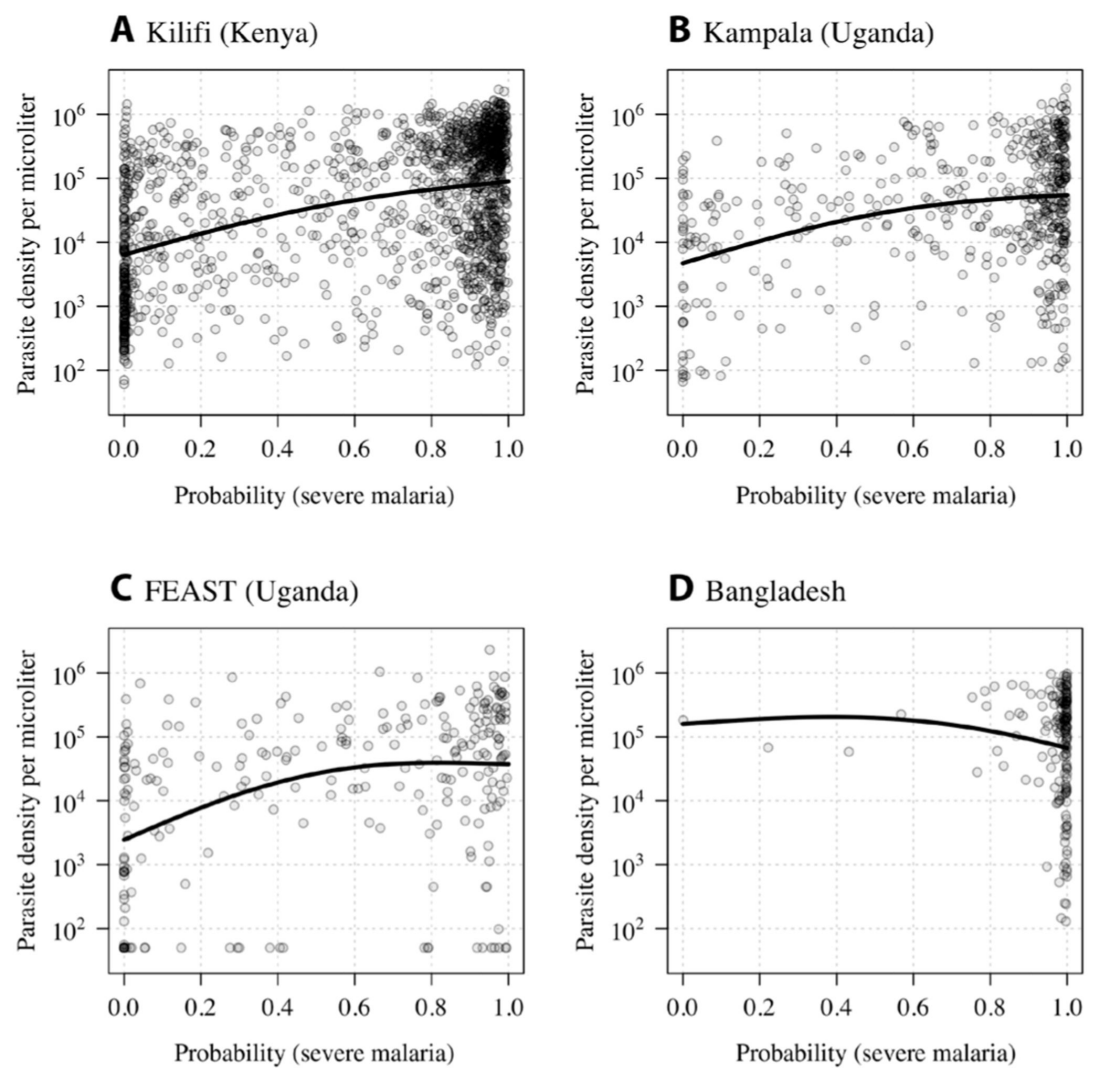
Admission parasite densities as a function of the probability of severe malaria under the Bayesian latent class model. Data from the FEAST trial include only the patients with a positive malaria rapid diagnostic test. The thick lines show the additive linear model fit (spline-based). (**A**) to (**D**) show the individual data for each study [(A) Kilifi cohort, (B) Kampala cohort, (C) FEAST trial, and (D) Bangladesh cohort].

We explored the relationship between the model-estimated probability of severe malaria and the admission parasite densities, admission hematocrit, the total white blood cell counts, and the blood culture positivity rates (i.e., cultures growing a likely pathogen). In the three African studies, parasite densities were between 12 and 16 times higher in patients with a high probability of severe malaria versus those with a low probability of severe malaria (Fig. 4, A to C). After adjusting for study differences, the parasite densities were estimated to be 12.8-fold higher (95% CI: 9.9 to 16.5) in severe malaria versus nonsevere malaria.

The admission hematocrits also highly correlated with the model-estimated probability of severe malaria (fig. S4, A to D). In the three African studies, children with a high probability of having severe malaria had median admission hematocrit values between 16 and 20% (FEAST: 19%, Kampala: 16%, and Kilifi: 20%). The hematocrit distributions in this group were unimodal (fig. S5, A to C). In contrast, the hematocrit distributions in patients with low probabilities of having severe malaria (<0.2) were strongly bimodal, with the majority of patients having higher hematocrits (the median hematocrit in this group were as follows: FEAST: 30%, Kampala: 12%, and Kilifi: 28%), but a substantial minority had low hematocrits of ~10% (fig. S5, D to F). The few Bangladeshi adults with a low probability of severe malaria also had higher hematocrits (fig. S4D).

Blood cultures were done for all 1400 Kenyan children and for 298 of the 332 (90%) Ugandan children in the FEAST study who had malaria parasitemia on admission. Overall, 51 and 35 patients, respectively, had positive blood cultures after removing likely contaminants. The probability of having severe malaria was highly predictive of the blood culture result, with an adjusted odds ratio of 0.43 (95% CI: 0.27 to 0.66, *P* = 0.0002) for a positive culture in patients likely to have severe malaria versus those unlikely to have severe malaria. This difference in blood culture positivity rates was also reflected in the total white blood cell counts (fig. S6). Across the four studies, after adjustment for age, in patients likely to have severe malaria (probability >0.5) compared to those unlikely to have severe malaria (probability <0.5), the odds ratio for having a total white count >15,000/μl was 0.50 (95% CI: 0.46 to 0.56, *P* = 10^−12^).

### Gene dose relationship for hemoglobin S and severe malaria

In the three studies in African children, the prevalence of both HbAS and HbSS (homozygous sickle cell anemia) was strongly inversely correlated with the model-estimated probability of severe malaria (Fig. 2, A to C). Pooling the three African studies and adjusting for differences in the estimated prevalence of severe malaria, the odds ratio for being classified as severe malaria (probability >0.5) for patients with HbAS relative to patients with HbAA was 0.25 (95% CI: 0.15 to 0.42, *P* = 10^−8^), and for patients with HbSS relative to patients with HbAA, the odds ratio was 0.08 (95% CI: 0.04 to 0.19, *P* = 10^−9^). Under an additive model of association, each additional hemoglobin S (HbS) allele was associated with an odds ratio for severe malaria of 0.27 (95% CI: 0.20 to 0.38, *P* = 10^−15^). This association between HbS genotypes and the marker-based severe malaria classification was highly concordant across the three studies.

## Discussion

The diagnosis of severe malaria in African children is imprecise (3, 4, 26). This is because it is difficult to distinguish clinically between severe illness caused by malaria from severe illness with incidental asymptomatic or uncomplicated malaria, because they share many clinical characteristics. This is a substantial problem for studies of one of the most important life-threatening infections in childhood. It dilutes and distorts the results of epidemiology (27), pathophysiology (3), genetic association (5), and therapeutic investigations (18). In areas of moderate and high levels of malaria transmission, asymptomatic parasitemia is very common. At any given time, a high proportion of children have detectable malaria parasitemia. Malaria parasitemia is therefore a sensitive but not specific indicator that malaria is the cause of illness (26). Other simple laboratory tests provide valuable diagnostic information. Thrombocytopenia is a common feature of all symptomatic malarias (6–8). A low platelet count therefore supports but does not prove that *P. falciparum* infection is the cause of the severe illness. We estimate that fewer than one in five patients with severe malaria will have platelet counts in the normal range (>150,000/μl). This analysis of data from large prospective studies of severe illness in African children shows that the combined measurement of platelet counts and the plasma concentration of the parasite protein *Pf*HRP2 substantially improve the specificity of the diagnosis of severe falciparum malaria.

A key strength of this work is that we can validate the discriminant power of the platelet count and the plasma *Pf*HRP2 concentration by comparing the prevalence of HbS genotypes (HbAS and HbSS) and the blood culture positivity rates across in the inferred subgroups. HbAS is the genotype that provides the strongest known protection against severe falciparum malaria (28, 29). The prevalence of HbAS was four times lower in children considered likely to have severe malaria compared with those considered less likely to have severe malaria. Note that HbAS also protects against complications of malaria, such as an increased risk of bacterial infections; thus, the HbAS prevalence in children with presumed bacterial infections isstill expected to be lower than that in the healthy population (30). Recent work suggests that the protective effect of HbAS may vary according to parasite genotype (31). It may be that the few HbAS individuals who have true severe malaria have parasite genotypes that can evade the HbAS defense mechanisms. Future work will assess the relationship between these probabilistic classifications and the parasite genotypes. Whereas people with the AS genotype are essentially hematologically normal, those with homozygous SS suffer from sickle cell anemia. This causes anemia and leukocytosis and may also cause thrombocytopenia. This could confound the use of full blood count data in the probabilistic assessment of severe malaria. However, the interpretation of the plasma *Pf*HRP2 concentration or other parasite biomass indicators should not be affected by sickle cell anemia. In this study, there was strong evidence for an additive effect between the number of HbS alleles and a decreased probability of severe malaria under the platelet/HRP2 model. This suggests that HbSS is strongly protective against a high parasite biomass (32). However, because sickle cell anemia crises may be severe and are often triggered by infections, it is possible that a low parasite biomass could trigger severe events. Whether or not this should be described as severe malaria is a semantic question (33). A recent in-depth analysis of the Ugandan cohort from Kampala showed that the children with HbSS considered to have severe malaria had a lower parasite biomass but higher levels of endothelial dysregulation (32). The main differential diagnosis in suspected severe malaria is bacterial sepsis. The relationship is complicated as severe malaria does predispose to bacterial sepsis (34). Blood culture has low sensitivity; however, we observed a threefold increase in positivity rates in the patients likely to have been misdiagnosed compared to those with a likely correct diagnosis.

The main limitation of this study is the absence of a gold standard diagnosis, and thus the reliance on parametric latent class models. The application of latent class models to multiple populations with varying disease prevalence provides a powerful framework for estimating the diagnostic accuracy of imperfect tests, but the model outputs rely on the validity of key assumptions, notably the underlying parametric models used for the marker distributions. We used log-normal distributions for the platelet count and plasma *Pf*HRP2 concentration in both true severe malaria and nonsevere malaria. Visual assessment of data plots suggest that this is a good approximation of the true underlying distributions. Each individual marker has its own limitations. Thrombocytopenia can be caused by other infections (e.g., severe arbovirus infection) and may occur in sepsis, although it is much less prevalent in sepsis than malaria (8). The main limitation of plasma *Pf*HRP2 as a marker of total parasite biomass is that it requires specialist measurement [although rapid point-of-care tests can be applied to plasma (35) and modifications are under development]. As plasma *Pf*HRP2 accumulates each asexual parasite cycle, an individual with a sustained low parasite multiplication rate at high parasite densities can have the same *Pf*HRP2 concentration as an individual with a fulminant infection and a high sequestered biomass (17). In addition, mutations in the *Pf*HRP2 gene causing changes in antigenicity may affect immunoassays, although these are currently rare outside of the Horn of Africa (36, 37).

In these large prospective series of patients hospitalized with a diagnosis of severe malaria, combining platelet counts and plasma *Pf*HRP2 concentrations provided good discrimination between true severe falciparum malaria and other severe illnesses, which are likely to be bacteremia in many of the cases, with concomitant incidental malaria. A major strength of this study is the combination of two measures that are mechanistically distinct: Platelet counts measure the host response to acute malaria illness, and *Pf*HRP2 measures the parasite biomass. We show that the combination of these markers provides a high level of discrimination between patients who were likely to have had true severe malaria and those with a different illness etiology. This proportion ranged from one-third in the FEAST trial, which intentionally studied children with severe malaria and other severe illnesses requiring fluid resuscitation, to >90% in Bangladesh, which is a low-transmission area where the diagnosis of falciparum malaria as the cause of illness is highly specific. These data suggest that about one-third of African children diagnosed with severe malaria have another cause of severe illness (5). Our results suggest that severe malaria with a normal platelet count is unusual, and a platelet count >150,000/μl in a child with suspected severe malaria should motivate further examination for a potential alternative cause of illness concomitant with prompt antimalarial treatment. The high diagnostic accuracy of the plasma *Pf*HRP2 concentration should motivate future work on simple modifications to the currently used *Pf*HRP2 rapid tests to estimate plasma *Pf*HRP2 at the bedside. Last, we recommend that future studies of severe falciparum malaria should include only children with platelet counts of ≤150,000/μl and plasma *Pf*HRP2 concentrations of ≥1000 ng/ml.

## Materials And Methods

### Study design

This study is a retrospective analysis of platelet counts and plasma *Pf*HRP2 concentrations in patients with severe febrile illness. We merged data from four separate studies, three of which were studies of severe malaria, and one was a study of fluid resuscitation in severe febrile illness (FEAST trial). The main goal of the analysis was to use Bayesian latent class modeling to estimate the proportion of misdiagnosed patients in the three severe malaria studies and to assess the operating characteristics of different cutoff values for the platelet count and *Pf*HRP2 concentrations for future clinical studies. For the analysis and reporting, we followed the Enhancing the Quality and Transparency of health Research (EQUATOR) guidelines for the reporting of diagnostic accuracy studies that use Bayesian latent class models [Standards for the Reporting of Diagnostic accuracy studies that use Bayesian Latent Class Models (STARD-BLCM); see the Supplementary Materials].

### Data

All the clinical studies were prospective studies of severe illness and had appropriate ethical approval. For the Kilifi cohort (Kenya), ethical approval was granted by the Kenya Medical Research Institute/National Ethical Review Committee in Nairobi, Kenya (SCC1192) and the Oxford Tropical Research Ethics Committee (OxTREC) in Oxford, UK (020-06). For the Kampala cohort (Uganda), institutional review boards for human studies at Makerere University and the University of Minnesota granted ethical approval for the study. Additional regulatory approval was obtained from the Uganda National Council for Science and Technology. The FEAST trial protocol was approved by ethics committees at Imperial College, London; Makerere University, Uganda; Medical Research Institute, Kenya; and National Medical Research Institute, Tanzania. For the Bangladesh cohort, ethical approval was given by the OxTREC and the Chittagong Medical College Hospital Ethics Committee. Reuse of existing, appropriately anonymized, human data does not require ethical approval under the OxTREC regulations.

### Bangladesh

We included data from observational studies in severe falciparum malaria conducted by the Mahidol Oxford Tropical Medicine Research Unit in Bangladesh between 2003 and 2019. These pooled data have been described previously (38). Malaria transmission is seasonal and of low intensity in this location. In brief, adults and children with asexual stage malaria parasites found by microscopy in thick and thin blood smears who met the WHO definition for severe malaria were enrolled in the study after written informed consent was obtained from the patient or an attending relative.

Criteria for severe malaria included coma (Glasgow Coma Scale Score <11 or Blantyre Coma Score <3), pulmonary edema, repeated convulsions (≥2 in 24 hours), severe anemia (hematocrit level <20% plus a parasite count >100,000 parasites/μl) or jaundice (bilirubin level >3.0 mg/dl in addition to a parasite count >100,000 parasites/μl), renal failure (serum creatinine level >3 mg/dl), hypoglycemia (blood glucose level <40 mg/dl), shock (systolic blood pressure <80 mm Hg with cool extremities), hyperparasitemia (peripheral asexual stage parasitemia >10%), hyperlactemia (venous plasma lactate >4 mM), and/or acidemia (venous plasma bicarbonate level <15 mM). Platelet counts and *Pf*HRP2 concentrations were jointly measured in a total of 172 patients. The majority of these patients received intravenous artesunate.

### Kilifi (Kenya)

The Kenyan case-control cohort has been described in detail previously (25). Severe malaria cases consisted of all children aged <14 years who were admitted with clinical features of severe falciparum malaria to the high dependency ward of Kilifi County Hospital between 11 June 1999 and 12 June 2008. Severe malaria was defined as a positive blood smear for *P. falciparum* along with the following: prostration (Blantyre Coma Score of 3 or 4), cerebral malaria (Blantyre Coma Score <3), respiratory distress (abnormally deep breathing), and severe anemia (hemoglobin <5 g/dl). The standard-of-care antimalarial treatment during this period was intravenous quinine.

### FEAST (Uganda)

FEAST was a multicenter randomized controlled trial comparing fluid boluses for severely ill children with shock (*n* = 3161) that was not specific to severe malaria (23). Children between 2 months and 12 years of age were eligible for the trial if they presented with a severe febrile illness complicated by impaired consciousness (prostration or coma), respiratory distress (increased labored breathing), or both, and with impaired perfusion, as evidenced by one or more of the following: a capillary refill time of 3 s or more, lower limb temperature gradient, weak radial pulse volume, or severe tachycardia (>180 beats per minute in children younger than 12 months of age, >160 beats per minute in children 1 to 5 years of age, or >140 beats per minute in children older than 5 years of age). Exclusion criteria were severe malnutrition, gastroenteritis, and noninfectious causes of shock (e.g., trauma, surgery, or burns).

Platelet counts and plasma *Pf*HRP2 concentrations were measured in 502 children in the Ugandan sites (Mulago National Referral Hospital, Mbale and Soroti Regional Referral Hospitals, and St. Mary’s Hospital, Lacor). The standard-of-care antimalarial treatment during this period was intravenous quinine.

### Kampala (Uganda)

The trial by Brand et al. (24) was an observational study of cerebral malaria and severe malarial anemia in Mulago Hospital, Kampala, Uganda. Children were enrolled if they were between 18 months and 12 years of age. Cerebral malaria was defined as coma (Blantyre Coma Score <3 or Glasgow Coma Scale Score <8) in the presence of asexual parasites by blood smear. Severe malarial anemia was defined as the presence of *P. falciparum* parasites in a blood smear in children with a hemoglobin level ≤5 g/dl. The standard-of-care antimalarial treatment during this study was intravenous quinine.

### Procedures

For the Kilifi cohort and the Ugandan sites in the FEAST trial, blood samples for bacterial cultures were collected in BD BACTEC Peds Plus bottles and processed with a BD BACTEC automated blood culture instrument (Becton Dickinson) for initial detection of bacteria in the blood. BD BACTEC-positive samples were subcultured on standard media by routine microbiological techniques. Either biochemical test kits (API, bioMérieux), serological tests, or both were used to confirm suspected pathogens. Good Clinical Laboratory Practice was audited by Qualogy, and external quality assurance was provided by the UK National External Quality Assessment service. The following organisms were considered as contaminants: *Bacillus* species, coryneforms, *Micrococcus* species, coagulase-negative *Staphylococcus*, and citrobacter.

In all four studies, plasma *Pf*HRP2 levels were quantitated using the previously published methodology (18). The lower limit of detection of the ELISA plasma assay is about 2 ng/ml. Patients in the Kilifi study and the FEAST trial were genotyped for the rs334 single-nucleotide polymorphism (HbS) using DNA extracted from fresh or frozen samples of whole blood as described in detail previously (25, 39).

### Statistical analysis

We fitted a series of Bayesian parametric latent class models to the available marker data (21). The key assumptions of the main models are summarized as follows:

1) The marginal distribution of each marker in each latent class is log-normal.
2) The marginal distribution of each marker in each latent class is the same across the different studies and countries.
3) Informative Bayesian priors on all parameters.

Sensitivity analyses relaxed assumptions 1 to 3 (using bivariate *t* distributions, only using data from African children, and using weakly informative priors). We compared models with and without correlation between the markers within each latent class. The models with correlation performed better and were more conservative.

In an initial exploratory analysis, we used the R package mclust (40) (Gaussian Mixture Modelling for Model-Based Clustering; this uses the expectation-maximization algorithm for parameter estimation) to estimate the number of latent components in the data (fig. S7). This suggested three major underlying clusters, one of which was only present in the FEAST trial (patients with no measurable plasma *Pf*HRP2 and normal platelet counts). We then used full Bayesian inference with informative priors to determine the distributions of the latent clusters. In the initial exploratory analysis, we also looked at the relationship between the peripheral parasite density and the two markers (fig. S8). This preliminary analysis showed that the malaria parasite density is a poor marker for discriminating between true severe malaria and other causes of illness.

It was not possible to obtain robust model fits when using the parasite count in combination with either the platelet count or the plasma *Pf*HRP2.

We used the posterior predictive distribution over each class in the two latent class model to estimate the sensitivity and specificity for any given threshold for the platelet count and the plasma *Pf*HRP2. Thus, for example, for a given platelet count threshold *x* (where *x* is an upper threshold value), the sensitivity is defined as 1 – *F*_SM_(*x*), and the specificity is defined as *F*_notSM_(*x*), where *F*_SM_ and *F*_notSM_ are the cumulative distribution functions for the platelet count in the severe malaria and not the severe malaria components, respectively.

The first set of models used data from the three severe malaria studies only (Kenyan children from Kilifi, Ugandan children from Kampala, and Bangladeshi adults). For each marker, the marginal distribution in each latent class (severe malaria versus not severe malaria) was assumed to be log-normal with the following informative priors (weakly informative): Means and standard deviations (SD) are given on the log 10 scale. For the mean platelet counts in the two latent classes, we used *N*(log_10_100,0.1) [weakly informative: *N*(log_10_100,0.5)] for the severe malaria class and *N*(log_10_200,0.1) [weakly informative: *N*(log_10_200,0.5)] for the not severe malaria class. The platelet count geometric mean of 100,000/μl versus 200,000/μl in severe malaria versus not severe malaria is informed by the results in (3) (an autopsy study in patients who had died with a diagnosis of cerebral malaria: The patients with evidence of parasite sequestration all had very low platelet counts compared to those who did not; we expect slightly lower platelet counts in all patients as the platelet count is a prognostic factor and has a diagnostic value).

For the mean *Pf*HRP2 in the two latent classes, we used *N*(log_10_2500,0.2) for severe malaria and *N*(log_10_500,0.2) for not severe malaria. The weakly informative prior used an SD of 0.5 instead of 0.2. The mean plasma *Pf*HRP2 values are informed from previous modeling of data from the AQUAMAT trial (18).

The SD on the log _10_ scale for each log-normal distribution was given an exponential prior, with the rate parameter set to 2 (i.e., a mean SD of 0.5 on the log _10_ scale). We used informative beta priors for the prevalence of severe malaria in the three studies: Beta(19,1) for Bangladesh, beta(14,6) for Kilifi, and beta(14,6) for Kampala [the weakly informative beta priors: beta(4,1), beta(2,1), and beta(2,1), respectively].

To assess the robustness of the model outputs, we performed three sensitivity analyses: (i) by changing the parametric form to a bivariate student-*t* distribution with 10 degrees of freedom (robustness to assumption 1), (ii) by fitting the model to data only from the studies in African children with severe malaria (Kampala and Kilifi; robustness to assumption 2 as the Bangladeshi adults could plausibly be different from African children in terms of parasite biomass and host response), and (iii) by using weakly informative priors (larger SDs around the prior mean values and weaker beta priors on the prevalence parameters).

For the second set of models, we included data from the FEAST trial (23). FEAST intentionally enrolled severely ill patients with and without malaria; thus, there is a subset of patients who are clearly distinct from all patients in the studies of severe malaria (this subset is characterized, on average, by a negative malaria blood slide, no measurable plasma *Pf*HPR2, and a normal platelet count). Thus, the latent class model needed to include a third component to fit the data. Under this model, the marker distributions were also assumed to be log-normal in the severe malaria class but were a mixture of two log-normal distributions for the not severe malaria class. We used the same priors for the second set of models with the addition of the following priors for the third additional component (which can be summarized as “severe illness with no evidence of acute malarial infection”): For the mean platelet count, we used *N*(log_10_300,0.1) for the second class of not severe malaria; for the mean *Pf*HRP2, we used *N*(log_10_1,0.2).

For the three-component model, Dirichlet priors were used for the prevalence parameters for each latent class: Dirichlet(19,1,0.1) for the Bangladesh study, Dirichlet(3,3,3) for the FEAST trial, and Dirichlet(14,7,1) for the Kampala and Kilifi studies. For all Dirichlet priors, the hyperparameters correspond to the severe malaria and the two not severe malaria classes in that order.

For patients with nonmeasurable plasma *Pf*HRP2 concentrations to be included in this analysis, we set all nonmeasurable concentrations to 1 ng/ml (about half the lower limit of detection of the ELISA assay). Patients who had nonmeasurable plasma *Pf*HRP2 concentrations but had parasite densities above 1000/μl were excluded from the analysis as these could represent *P. falciparum* parasites with *HRP2/3* gene deletions. The median platelet count in these samples was 142,000/μl (range: 28,000 to 808,000/μl).

All posterior distributions were estimated using Monte Carlo methods. All models were implemented in the Stan language (41). For each model, we ran four independent chains for 5000 iterations, discarding half for burn-in. Convergence was checked by visually assessing traceplots. Convergence problems due to “label switching” were avoided by using parameter constraints: The mean value of each likelihood distribution was set as increasing (for the platelet count) or decreasing (for the *Pf*HRP2 and parasite density). This uses the ordered parameter class in Stan. The Stan language does not support discrete parameters; however, posterior distributions of latent class models can be sampled by using the LogSumExp trick, computing the marginal likelihood over the different class combinations.

## Supplementary Material

**Severe malaria insights**

Diagnosis of children with severe malaria caused by infection with *Plasmodium falciparum* has been difficult in hightransmission settings because of the high coincidence of malaria with other febrile illnesses. Watson *et al*. analyzed data from >2000 severely ill children and adults in low-transmission (Bangladesh) and high-transmission (Kenya and Uganda) settings. By fitting Bayesian latent class models using a combination of platelet counts and plasma concentrations of *P. falciparum* histidine-rich protein 2 (*Pf*HRP2), they estimated that detection of #150,000 platelets/ μl and a plasma *Pf*HRP2 concentration of #1000 ng/ml had a sensitivity of about 74% and a specificity of about 93% in identifying severe malaria. These findings revealed that a proportion of children enrolled in several malaria clinical studies in high-transmission settings have severe febrile illness caused by other types of pathogens.

Checklist.pdf

Checklist.zip

Fig S1 to S8, Table S1

## Figures and Tables

**Table 1:** Patient characteristics across four studies. For age, parasite densities, platelet counts, total white blood cell counts, and *Pf*HRP2 concentrations, we show the median values with IQRs in parentheses. No HbS genotyping was done for Bangladeshi patients, because HbS is absent in this population.

	Kilifi (Kenya)	Kampala (Uganda)	FEAST (Uganda)	Bangladesh
*n*	1400	492	559	171
Age (years, IQR)	2.4 (1.4–3.7)	3.3 (2.2–4.6)	2.0 (1.2–3.3)	30 (23–45)
Proportion parasite positive (%)	100	100	59.4	100
Parasite density * (per μl, IQR)	69,824 (6099–316,350)	42,530 (10,635–198,540)	37,600 (3640–153,680)	148,874 (23,550–348,540)
Platelet count (×10^3^/μl, IQR)	111 (64–215)	96 (49–170)	165 (75–326)	50 (27–139)
*Pf*HRP2 (ng/ml, IQR)	2207 (419–5072)	1838 (588–4097)	175 (0–1953)	2667 (1083–6128)
White blood cell count (/μl, IQR)	12.6 (8.9–19)	10.4 (7.5–15.3)	12.0 (8.4–18.7)	9.0 (6.9–11.0)
Mortality (%)	11.1	6.7	11.4	26.9
HbAS (*n*, %)	41 (2.9)	4 (0.8)	46 (8.2)	–
HbSS (*n*, %)	7 (0.5)	23 (4.7)	21 (3.8)	–

* For the FEAST trial, parasite densities refer only to patients with a positive rapid diagnostic test.

## Data Availability

All data associated with this study are present in the paper or the Supplementary Materials. All data and code used in the analysis are fully available on a GitHub repository at https://github.com/jwatowatson/SevereMalariaDiagnosis (42). The latest version has been archived on Zenodo at https://zenodo.org/badge/latestdoi/412716066. All figures can be reproduced via the R Markdown script provided on the GitHub repository.
