# Supplementary material for: Improving the diagnosis of severe malaria in African children using platelet counts and plasma PfHRP2 concentrations
Source: Sci Transl Med. Author manuscript; Available in PMC 2022 Sep 21. (PMC7613613; doi:10.1126/scitranslmed.abn5040)
Supplement: Checklist.zip [file EMS153592-supplement-Checklist_zip.zip › scitranslmed.abn5040_equator_stard_blcm_checklist.docx]

|  | **Section & Topic** | **No** | **Item** | **Reported on page #** |
| --- | --- | --- | --- | --- |
|  |  |  |  |  |
|  | **TITLE OR ABSTRACT** |  |  |  |
|  |  | **1** | Identification as a study of diagnostic accuracy, using at least one measure of accuracy (such as sensitivity, specificity, predictive values, or AUC) **and Bayesian latent class models** | 2 |
|  | **ABSTRACT** |  |  |  |
|  |  | **2** | Structured summary of study design, methods, results, and conclusions  (for specific guidance, see STARD for Abstracts) | 2 |
|  | **INTRODUCTION** |  |  |  |
|  |  | **3** | Scientific and clinical background, including the intended use and clinical role of the **tests under evaluation** | 3-4 |
|  |  | **4** | Study objectives and hypotheses, **such as estimation of diagnostic accuracy of the tests for a defined purpose through BLCM** | 5 |
|  | **METHODS** |  |  |  |
|  | *Study design* | **5** | Whether data collection was planned before the **tests** were performed (prospective study) or after (retrospective study) | 16 |
|  | *Participants* | **6** | Eligibility criteria **and description of the source population** | 16-18 |
|  |  | **7** | On what basis potentially eligible participants were identified  (such as symptoms, results from previous tests, inclusion in registry) | 16-18 |
|  |  | **8** | Where and when potentially eligible participants were identified (setting, location and dates) | 16-18 |
|  |  | **9** | Whether participants formed a consecutive, random or convenience series | 16-18 |
|  | *Test methods* | **10** | **Description of the tests under evaluation**, in sufficient detail to allow replication, **and/or cite references** | 18-19 |
|  |  | **11** | Rationale for choosing the **tests under evaluation in relation to their purpose** | 3-4 |
|  |  | **12** | Definition of and rationale for test positivity cut-offs or result categories of **the tests under evaluation**, distinguishing pre-specified from exploratory | N/A |
|  |  | **13** | Whether clinical information was available to the performers or readers of **the tests under evaluation** | N/A |
|  | *Analysis* | **14a** | **BLCM model** for estimating measures of diagnostic accuracy | 19-22 |
|  |  | **14b** | **Definition and rationale of prior information and sensitivity analysis** | 19 |
|  |  | **15** | How indeterminate results **of the tests under evaluation** were handled | N/A |
|  |  | **16** | How missing data **of the tests under evaluation** were handled | N/A |
|  |  | **17** | Any analyses of variability in diagnostic accuracy, distinguishing pre-specified from exploratory |  |
|  |  | **18** | Intended sample size and how it was determined | N/A |
|  | **RESULTS** |  |  |  |
|  | *Participants* | **19** | Flow of participants, using a diagram | 7 |
|  |  | **20** | Baseline demographic and clinical characteristics of participants | 7 |
|  |  | **21** | **Not applicable: the distribution of the targeted conditions is unknown, hence the use of BLCM** |  |
|  |  | **22** | Time interval and any clinical interventions between **the tests under evaluation** | N/A |
|  | *Test results* | **23** | Cross tabulation of the **tests’ results (or for continuous tests results their distribution by infection stage)** | Supplementary Fig S7-S8 |
|  |  | **24** | Estimates of diagnostic accuracy **under alternative prior specification** and their precision (such as 95% **credible/probability intervals**) | Supplementary Fig S1 |
|  |  | **25** | Any adverse events from performing **the tests under evaluation** | N/A |
|  | **DISCUSSION** |  |  |  |
|  |  | **26** | Study limitations, including sources of potential bias, statistical uncertainty, and generalisability | 14 |
|  |  | **27** | Implications for practice, including the intended use and clinical role of **the tests under evaluation in relevant settings (clinical, research, surveillance etc.)** | 15 |
|  | **OTHER INFORMATION** |  |  |  |
|  |  | **28** | Registration number and name of registry | NA |
|  |  | **29** | Where the full study protocol can be accessed | NA |
|  |  | **30** | Sources of funding and other support; role of funders | 26 |
|  |  |  |  |  |

STARD - BLCM

STARD-BLCM stands for “Standards for the Reporting of Diagnostic accuracy studies that use Bayesian Latent Class Models” and is a modification of the STARD statement (which was recently updated to STARD2015). STARD-BLCM aims to facilitate improved quality of reporting for diagnostic accuracy studies that use Bayesian latent class models in the absence of a reference standard. The proposed modifications are relevant to both Bayesian and frequentist estimation methods but the focus is on the former.

More information for STARD (STARD2015) can be found at: [http://www.equator-network.org/reporting-guidelines/stard](http://www.equator-network.org/reporting-guidelines/stard/)

More information for STARD-BLCM can be found at: [http://www.equator-network.org/reporting-guidelines/stard-blcm](http://www.equator-network.org/reporting-guidelines/stard-blcm/)
