# Supplementary material for: Improving the diagnosis of severe malaria in African children using platelet counts and plasma PfHRP2 concentrations
Source: Sci Transl Med. Author manuscript; Available in PMC 2022 Sep 21. (PMC7613613; doi:10.1126/scitranslmed.abn5040)
Supplement: Fig S1 to S8, Table S1 [file EMS153592-supplement-Fig_S1_to_S8__Table_S1.pdf]

Science Translational Medicine
AAAS

Supplementary Materials for

Improving the diagnosis of severe malaria in African children using platelet counts and plasma *Pf* HRP2 concentrations

James A. Watson *et al.*

Corresponding author: James A. Watson, jwatowatson@gmail.com

Sci. Transl. Med. **14**, eabn5040 (2022)
DOI: 10.1126/scitranslmed.abn5040

The PDF file includes:

Figs. S1 to S8
Table S1

Other Supplementary Material for this manuscript includes the following:

EQUATOR STARD-BLCM Checklist
MDAR Reproducibility Checklist

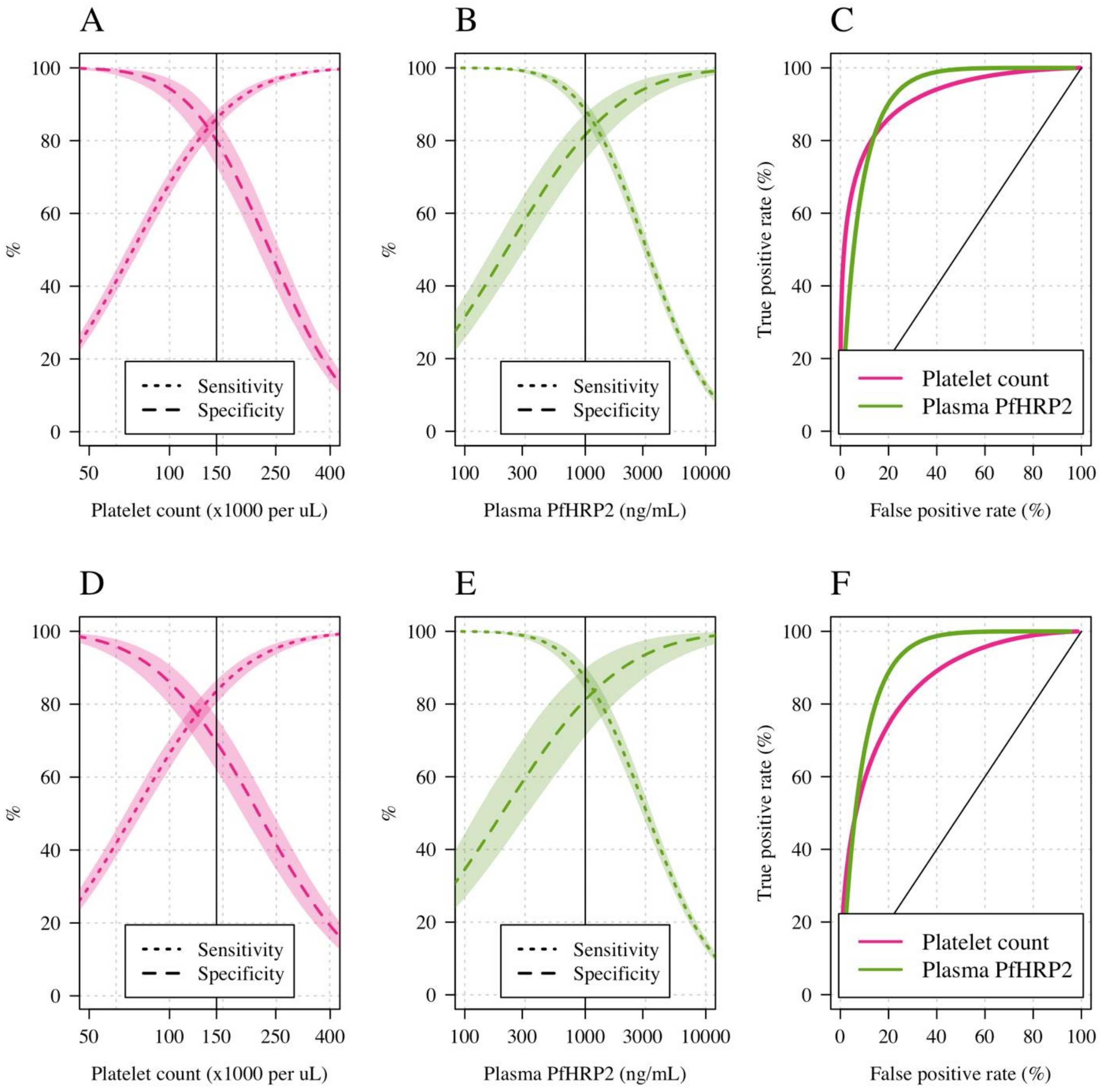

Figure S1: Receiver operating characteristics for the two main sensitivity analyses performed. Panels A-C are the same as in Fig. 1 main text but estimated under a model using bivariate *t*-distributions instead of bivariate normal distributions. Panels D-F shows the same panels as in Fig. 1 main text but with weakly informative priors (see Methods). When using a bivariate *t*-distribution, the model estimates a higher specificity for the platelet count but from visual inspection of the patients for whom the posterior probability changes considerably, we decided that this is most likely to be wrong, reflecting prior beliefs that the plasma *Pf*HRP2 is more specific given that thrombocytopenia can be caused by other diseases or conditions such as sickle cell anemia.

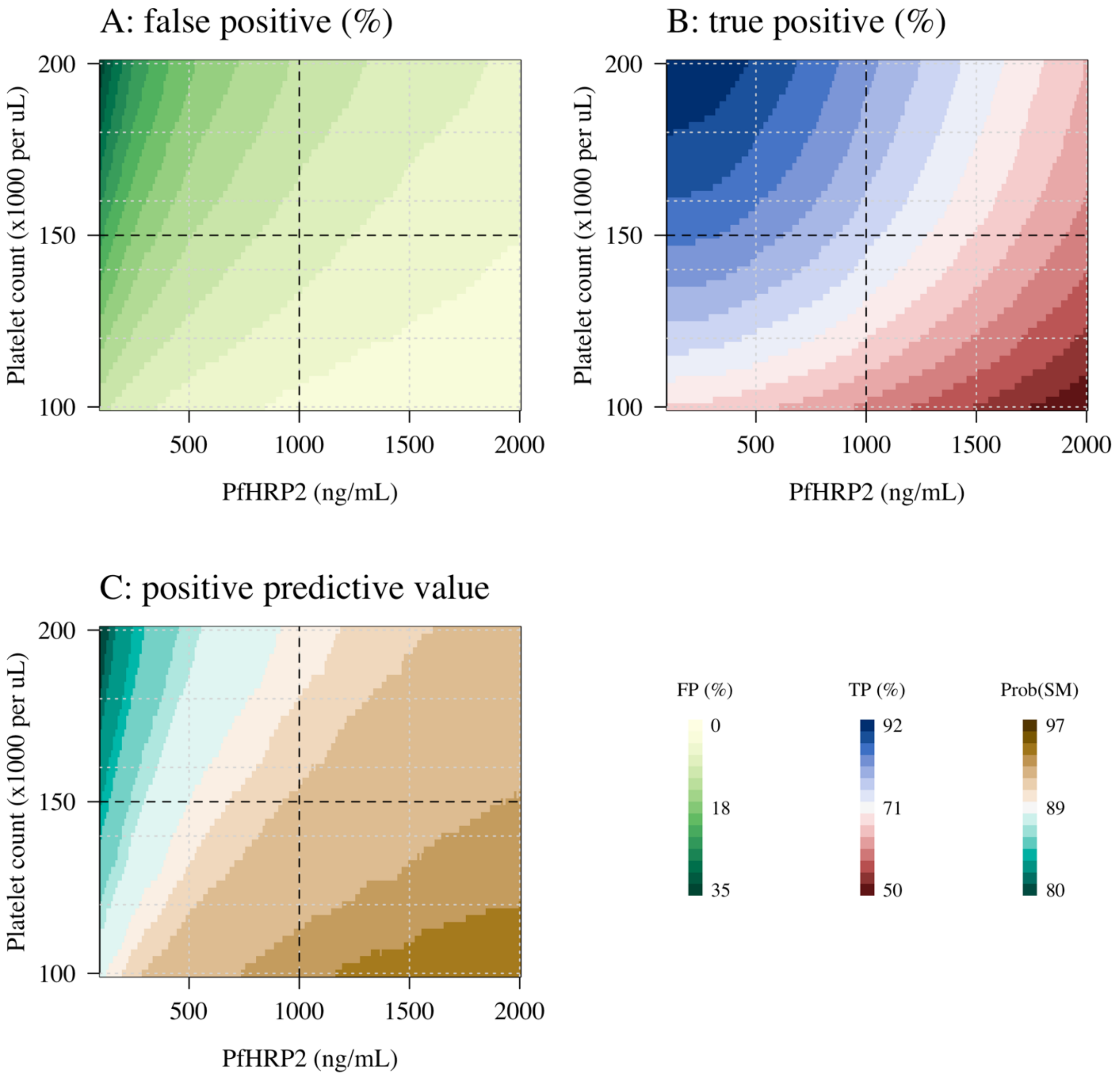

Figure S2: The estimated operating characteristics for the platelet count and the plasma *Pf*HRP2 concentration when used in combination to diagnose severe falciparum malaria. A-B: false positive and true positive rates as a function of the joint platelet count and *Pf*HRP2 thresholds. C: the positive predictive value assuming 60% prevalence of severe malaria in target population (hospitalized children with clinical features consistent with severe malaria). TP: true positive rate; FP: false positive rate. Prob(SM): posterior probability of severe malaria under the model.

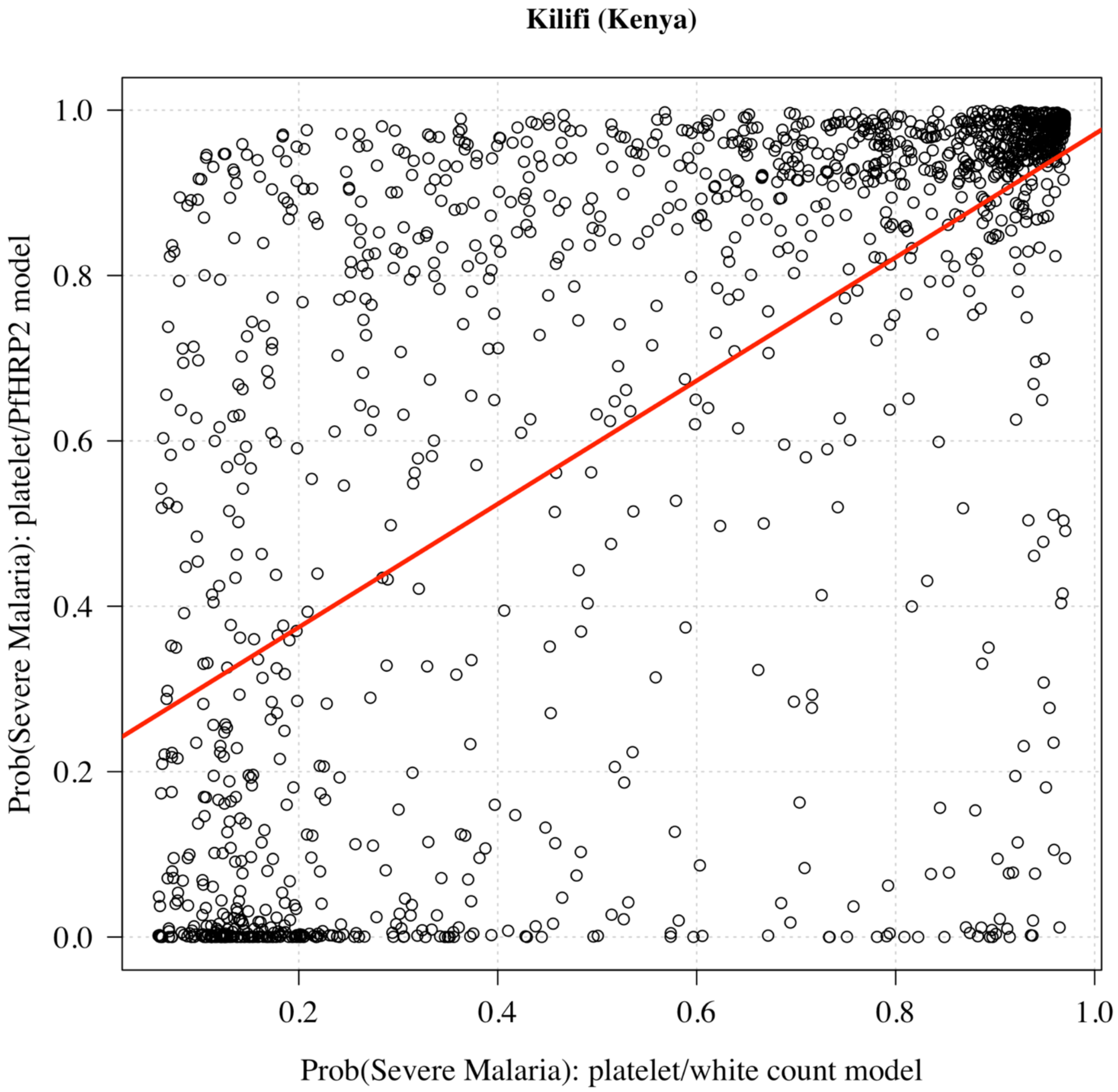

Figure S3: Estimated probabilities of severe malaria under the previously published platelet/white count model (ref 5 in main text) and those under the platelet/HRP2 model for 1,400 Kenyan children who had both plasma *Pf*HRP2 measurements and platelet counts.

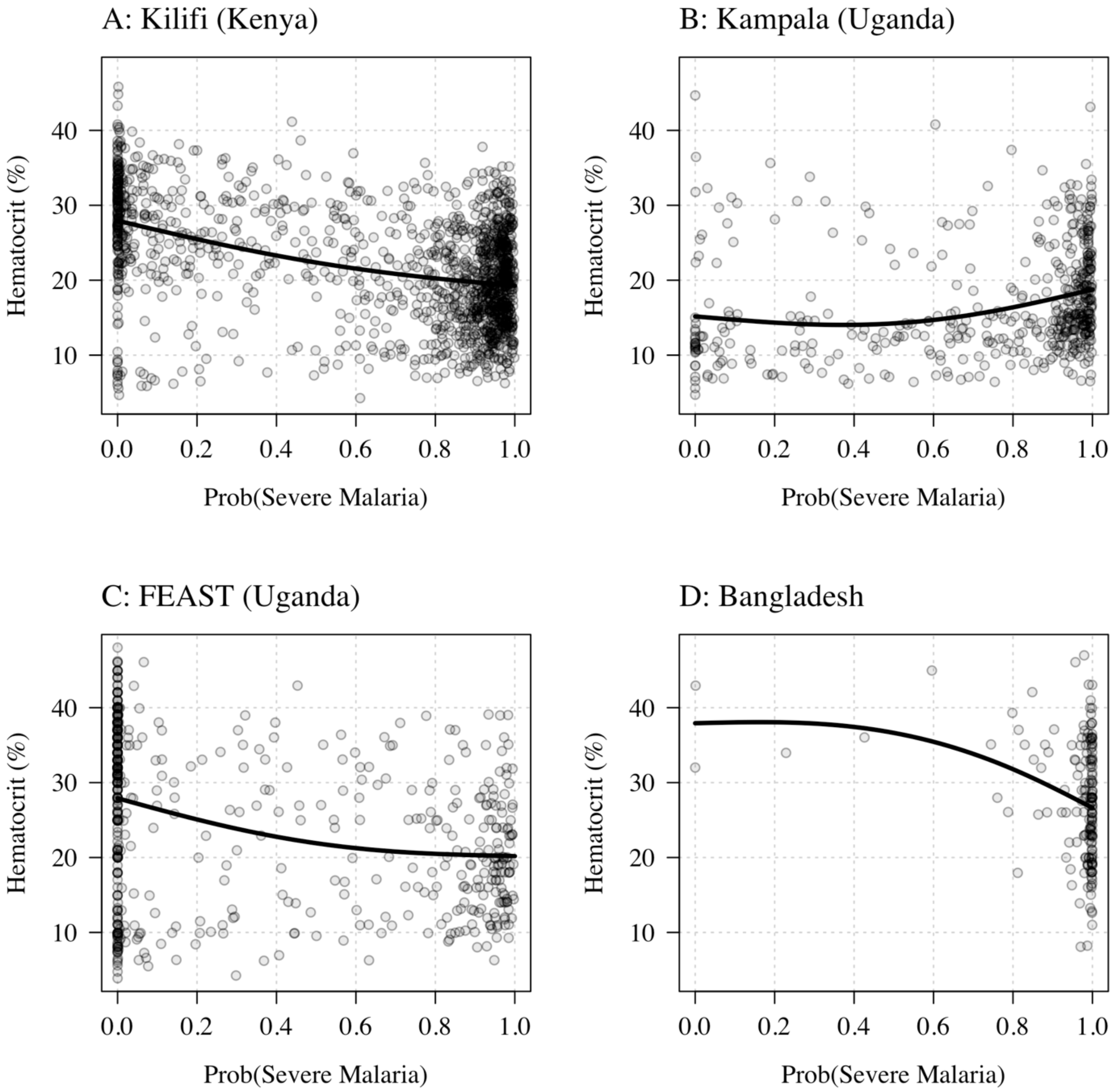

Figure S4: Admission hematocrits as a function of the probability of severe malaria under the Bayesian latent class model (model probabilities based on the platelet counts and the plasma *Pf*HRP2 concentrations).

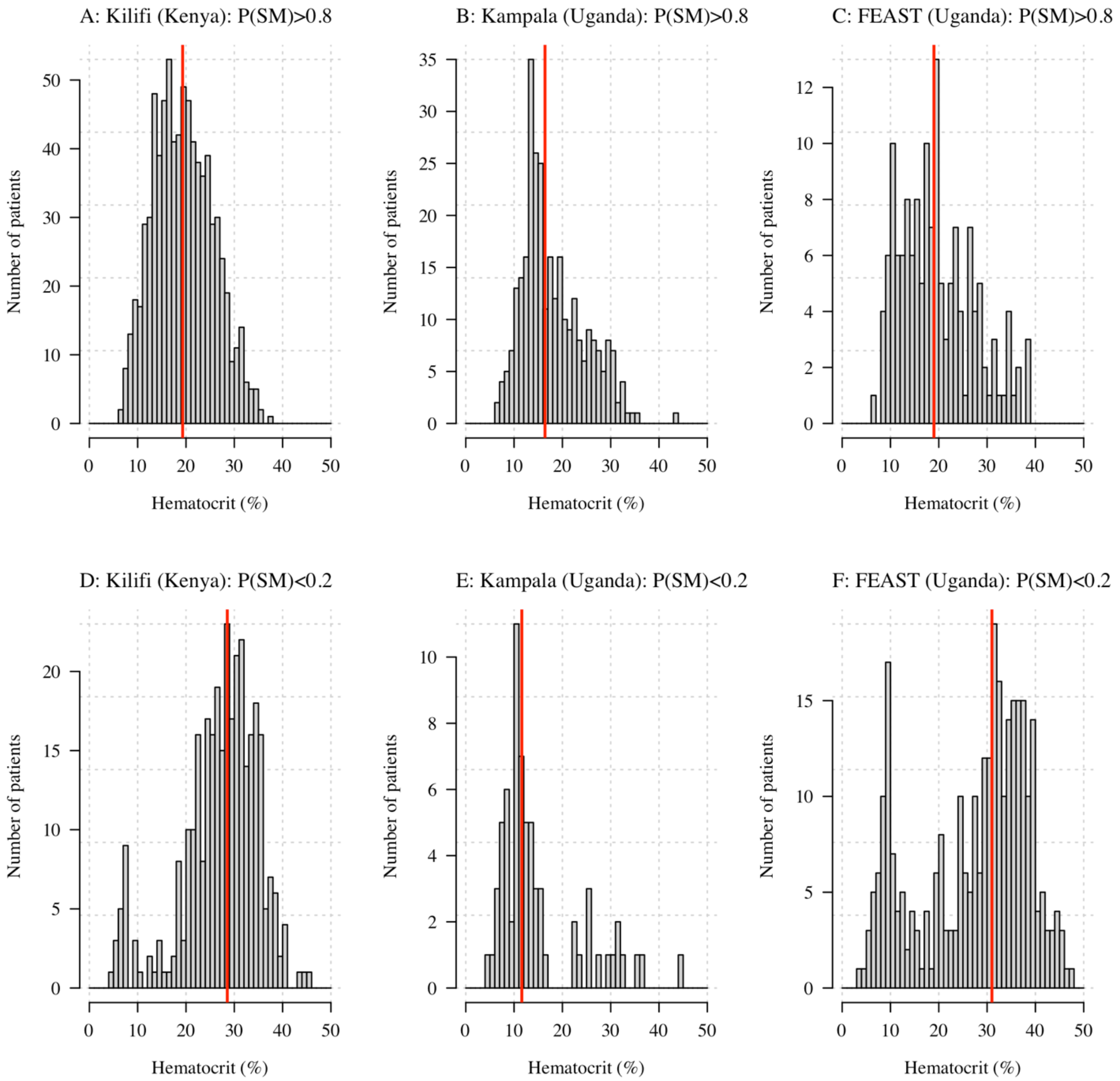

Figure S5: Distribution of hematocrits in African children with a high (>0.8: panels A-C) and low (<0.2: panels D-F) probability of having severe malaria. The vertical red lines show the median values.

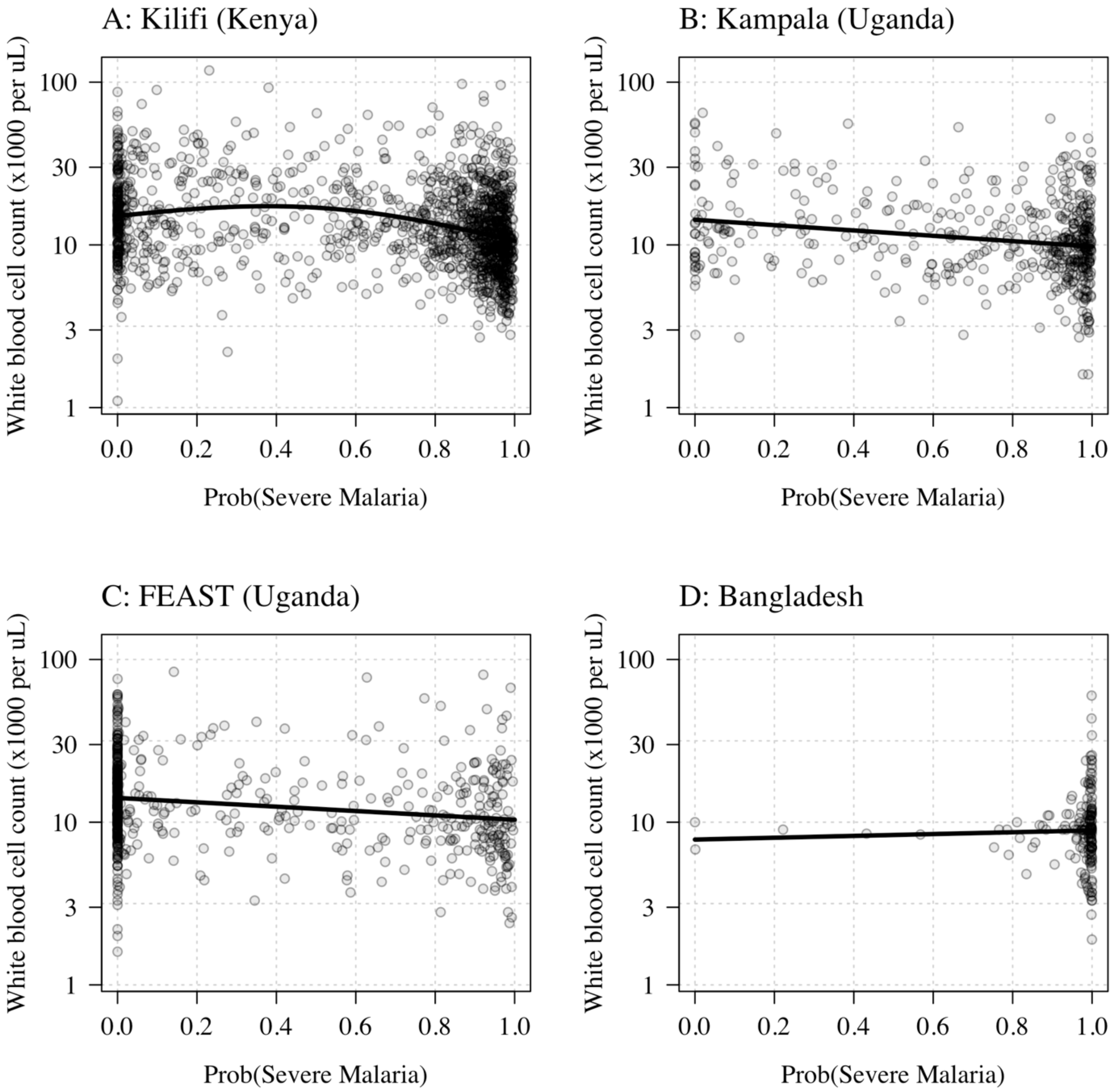

Figure S6: Total white blood cell counts (logarithmic scale) as a function of the model estimated probability of severe malaria (based on platelet counts and *Pf*HRP2 concentrations). The thick black lines show the overall trends. (A: Kilifi cohort; B Kampala cohort; C: FEAST trial; D: Bangladesh cohort).

(A: Kilifi cohort; B Kampala cohort; C: FEAST trial; D: Bangladesh cohort)

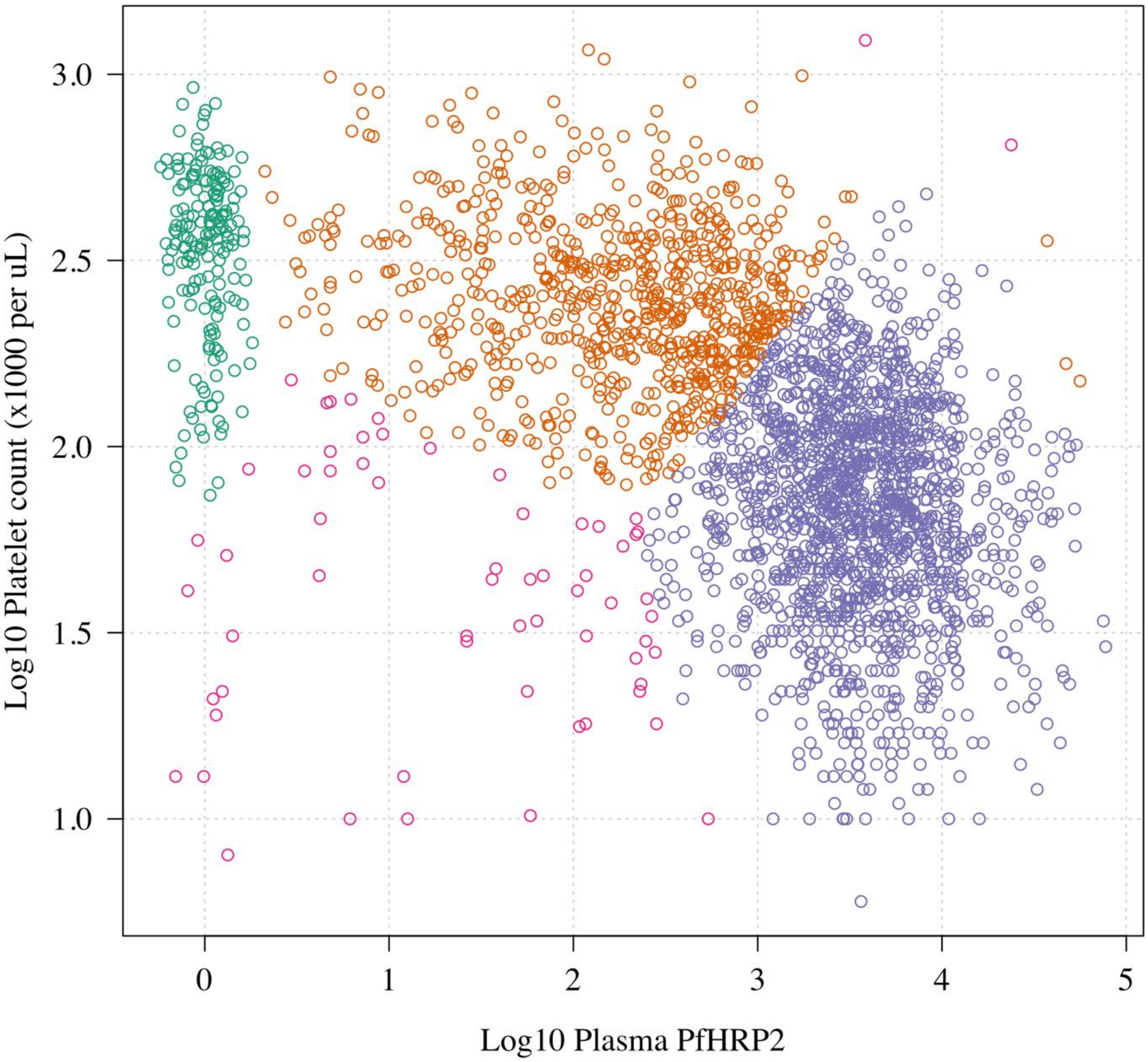

Figure S7: Bayesian clustering of all data using the *mclust* package (ref 40 in main text). This identifies 3 major components (green/orange/purple) and a small minority of patients who have both low platelet counts and low plasma *Pf*HRP2 concentrations (most of these are from the FEAST trial, pink).

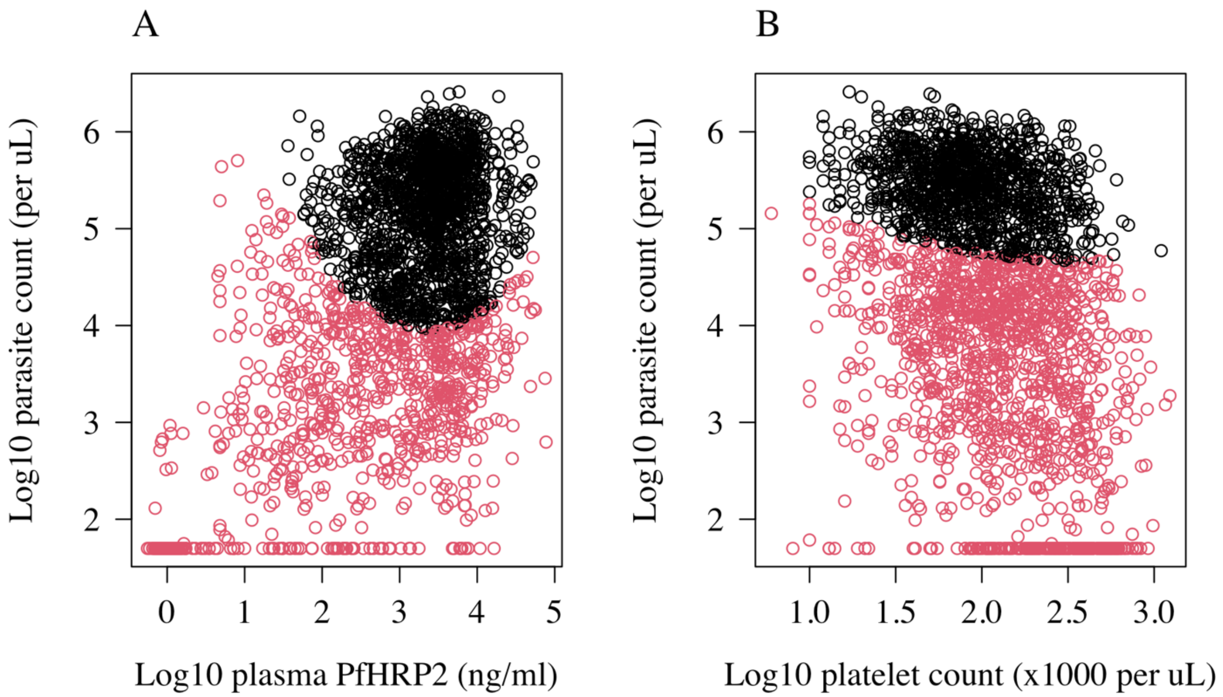

Figure S8: Bayesian clustering of the *Pf*HRP2/Parasite density data (A) and the platelet count/parasite density data (B) using the *mclust* package (ref 40 in main text). In both cases this identifies only 2 major components (red/black) with poor fits to the data.

	Kilifi (Kenya)	Kampala (Uganda)	FEAST (Uganda)	Bangladesh
Coma (%)	74	88	44	98
No coma (%	65	64	34	97
Hb < 5g/dL	86	68	41	100
Hb > 5g/dL	65	86	34	97

Table S1: Mean posterior estimates of the proportion of `true' severe malaria in for patients with or without coma, and patients with or without severe anemia (hemoglobin level less than 5 g/dL); Hb: hemoglobin.
